# Skills attained by infants with congenital Zika syndrome: Pilot data from Brazil

**DOI:** 10.1371/journal.pone.0201495

**Published:** 2018-07-26

**Authors:** Anne C. Wheeler, Camila V. Ventura, Ty Ridenour, Danielle Toth, Lucélia Lima Nobrega, Lana Claudia Silva de Souza Dantas, Camilla Rocha, Donald B. Bailey, Liana O. Ventura

**Affiliations:** 1 RTI Center for Newborn Screening, Ethics, and Disability Studies, RTI International, Research Triangle Park, NC, United States of America; 2 Rehabilitation Center “Menina dos Olhos”, Altino Ventura Foundation (FAV), Recife, Brazil; ESIC Medical College & PGIMSR, INDIA

## Abstract

The recent Zika outbreak and its link to microcephaly and other birth defects in infants exposed in utero have garnered widespread international attention. Based on the severity of birth defects the extent of impairment in these infants is expected to be profound; however, virtually nothing is known regarding the developmental and behavioral sequela of congenital Zika syndrome. This pilot study collected parent-reported patterns of development and sleep in 47 infants with confirmed congenital Zika syndrome who are being followed for clinical services at the Altino Ventura Foundation (FAV) in Recife, Brazil. With assistance from clinicians at FAV, caregivers completed Brazilian Portuguese versions of the Ages and Stages Questionnaire, 3^rd^ edition (ASQ-3) and the Brief Infant Sleep Questionnaire (BISQ). All infants were between 13–22 months of age at the time of the assessment. At 16 months of age, none of the children displayed age appropriate developmental skills. Most (~ 75%) mastered some communication and gross motor skills at around a 6–8-month level. Communication and gross motor skills were relative strengths for the sample, while problem-solving and fine motor skills were relative weaknesses. Sleep was noted to be a problem for around 18% of the sample. In utero exposure to the Zika virus will have lifelong consequences for affected children and their families. Understanding the developmental and behavioral trajectories of affected infants will help identify appropriate family supports to improve quality of life.

## Introduction

The 2016 outbreak of Zika infection in northeastern Brazil resulted in an epidemic of what is now known as congenital Zika syndrome (CZS). Over 3,000 babies in Brazil have diagnosed or suspected CZS. The initial criteria for a diagnosis of CZS in children is exposure to the Zika virus (ZIKV) while in utero, with expression of some of the common features of CZS, including microcephaly [1], central nervous system (CNS) damage [2], subcortical calcifications, ocular damage resulting in macular scarring and focal pigmentary retinal mottling, congenital contractures, and hypertonia extrapyramidal involvement [3]. Electromyography and brain imaging studies have identified neurologic lesions involving the central and peripheral nervous systems resulting in hip dysplasia, subluxation of large joints, abnormal posturing of extremities, conductive hearing loss, and abnormalities of the retina and optic nerve. Early reports of neurodevelopment in 19 infants suggest severe to profound cognitive impairments [4]. While it is expected that these children will have normal lifespans, they are likely to be severely limited in their functional skills and require constant care [5]. However, almost nothing is known regarding skill attainment in children with CZS, or the factors that contribute to variability in outcomes. This paper describes findings from a pilot study of early developmental profiles of infants with CZS, with a focus on the skills attained by 16 months of age in the areas of very early problem solving, communication, motor, and social-emotional development. We also explore potential individual and family predictors of better outcomes for infants with CZS to inform intervention and treatment development.

## Materials and methods

### Study design and participants

This study took place at the Altino Ventura Foundation (FAV) in Recife, Brazil, in spring 2017, when the cohort of infants affected during the 2015–2016 ZIKV outbreak were around 16 months of age (standard deviation [SD] = 3, range 12–24 months). Forty-seven mothers of infants (25 girls, 27 boys) with CZS who participated in weekly therapy sessions at FAV were interviewed about their child’s development. All infants were either born with birth defects consistent with CZS (e.g. microcephaly) or were known through parental report to have been exposed to Zika in utero and were subsequently referred to FAV for early intervention services. All infants had serologic confirmation of CZS via the antibody-capture enzyme linked immunosorbent assay (MAC-ELISA) performed on cerebrospinal fluid (CSF). This study was approved by the Institutional Review Board at the Altino Ventura Foundation 2180162. Parents provided written consent for themselves and their child to participate in this study.

The Brazilian versions of the Ages and Stages Questionnaire, 3^rd^ edition (ASQ-3) [6,7] and the Brief Infant Sleep Questionnaire (BISQ) [8] were administered by trained data collectors to assess the child’s overall sleep quality as well as level of skill attainment in the areas of communication, gross motor, fine motor problem-solving, and social-emotional development. Because of the low literacy level of many of the parents in this study, members of the research team read all items aloud to the respondents.

A review of each child’s medical records was conducted to obtain information about potential predictive variables such as maternal age at birth, timing of Zika exposure, other prenatal exposures (e.g., alcohol, tobacco), severity of microcephaly at birth, severity of visual impairment, and gestational age. Binocular visual impairment is based on results of the Teller Acuity Cards [9, 10] for pediatric visual acuity. Table 1 lists the demographics of the sample.

**Table 1:** Demographics of sample.

N	47
Sex	
Female	24 (51.1%)
Male	23 (48.9%)
Microcephaly at birth*	43 (93.8%)
Microcephaly classification	
Severe (<−3)	29 (67.4%)
Mild (>−3<−2)	14 (32.6%)
Mean head circumference at birth (SD; range)	28.6 cm (1.84; 23–32.5)
Mean maternal age at birth (SD; range)	26.9 (7.5; 16–42)
Trimester Zika symptoms	
1	15 (32%)
2	12 (25%)
3	3 (6%)
Unknown	14 (30%)
Mean gestational age at birth in weeks (SD; range)	37.8 (2.1; 31.4–40.6)
Prematurity	11 (23.4%)
Mean birthweight (SD; range)	2.727 kg (0.52; 1.66–3.93)
Mean length (SD; range)	45.20 cm (3.54; 32–54)
Binocular severity of Visual Impairment (VI) (n = 43):	16.3% None 7.0% Low vision 34.9% Mild VI 41.9% Severe VI
Hearing impairment (n = 29 tested)	17%
Family Income**	2% (1) < 1 minimum wages 53% (25) 1 minimum wages 28% (13) 1–2 minimum wages 4% (2) 2–3 minimum wages 13% (6) Unknown
Alcohol exposure	11% (5)
Tobacco exposure	4% (2)
Arthogryposis	11% (5)
Clubfoot	15% (7)
Crooked foot	2% (1)
Suropodalic orthosis	85% (40)
Hip spacer	11% (5)
Hypertonia	81% (38)
Hip dislocation	24% (11)

* One infant had no record of head circumference measurement at birth; two infants were born premature and were not microcephalic at birth, but were microcephalic at their first clinical visit (~ 2 months of age).

**Family income in Brazil calculated based on the number of minimum wages earned within the household. 1 minimum wage is estimated to be an annual salary of around 3,672 US dollars. All families live below the national poverty line.

## Data analysis

Descriptive analyses were conducted to identify the number and percent of infants who had mastered each developmental skill assessed by the ASQ-3 6- and 8-month level skills in the overall sample and separately by gender. Gender differences were tested using analysis of variance (ANOVA). Cronbach’s alpha was run to determine the internal consistency of the ASQ-3 scales for this sample. Pearson correlations were used to summarize variables associated with better or worse outcomes. Because at this early stage of investigation Type II errors would be worse than Type I and because of the small sample size, statistical tests were conducted at the p < .05 level and heterogeneity in variances was not accounted for in ANOVAs.

## Results

**Fig 1:**
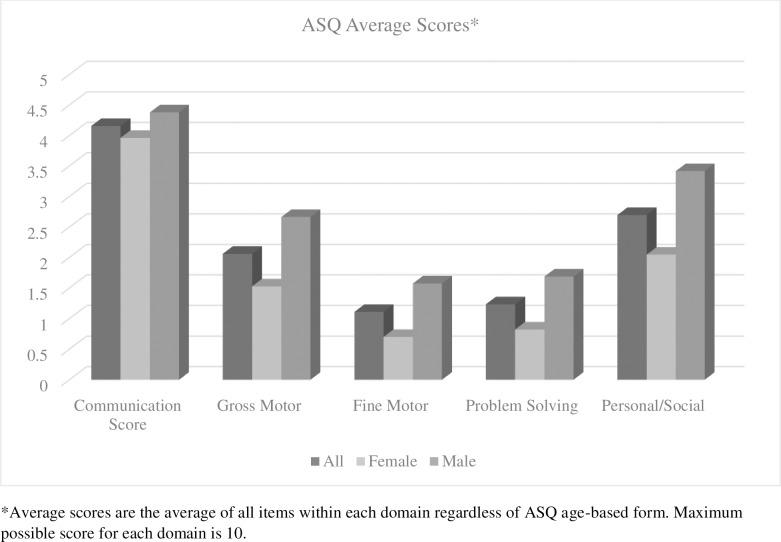
Average scores on ASQ domains by gender.

At the time of this assessment, none of the infants were able to demonstrate any age-appropriate skills. All infants required administration of the ASQ-3 form for either 6, 8, or 9 months of age. Because the form administered was much younger than the children’s chronological age, floor effects for standard scores would reduce the meaningfulness of ASQ reporting. Items were scored according to the metrics provided by the ASQ-3: 10 for “Mastered,” 5 for “Emerging,” 0 for “Not Yet.” Because more than one age version of the ASQ-3 was administered to some, but not all children, we took the best score for each item in each domain across the different versions administered, then averaged the scores for each participant for each domain. Total average scores for each domain for the full sample, and for males only and females only are illustrated in Fig 1. Correlations between developmental outcomes and individual or family variables hypothesized to be related to outcomes are summarized in Table 2. Findings for each domain are summarized below.

**Table 2:** Correlations between individual/family variables and developmental outcomes.

	Communication Score Sum	Gross Motor Sum	Fine Motor Sum	Problem Solving Sum	Personal/ Social Sum
Gestational age	0.141	0.008	0.088	0.091	−0.105
Birth weight	0.281^*^	0.171	0.128	0.255^a^	0.228^a^
Head circumference at birth	0.104	0.119	0.094	0.208^a^	0.174
Trimester of exposure	−0.005	−0.008	0.064	0.191	−0.013
Sex	0.048	0.340^*^	0.250^a^	0.218^a^	0.248^a^
Maternal age at birth	0.170	0.179	0.132	0.119	0.245
Severity of visual impairment: binocular test	-0.016	−0.025	−0.350^*^	−0.247	−0.214

N = 37–47. Tabulated values are Pearson correlations.

^a^ p < .10.

*p < .05.

Notes: p-value for birthweight with problem solving is .052.

p-value for alcohol with gross motor sum is .051.

p-value for sex with fine motor sum is .055.

### Communication

Relative to other assessed areas of development, communication skills were a relative strength. Most infants were reported to communicate using sounds, including loud squealing sounds (72%) and noises during play (77%). Most infants also responded to sounds in their environment, including responding to caregiver’s voice (72%), looking in the direction of sounds (83%), and responding to “no” (57%). Around one-quarter of infants engaged in back-and-forth interactions using vocalizations. However, very few infants were producing meaningful sounds, and none were able to follow one-step directions or engage in play activities without direction (although for a couple of the children, these skills were emerging). Birth weight was the only variable associated with Communication scores (0.28, p < .05).

### Gross motor skills

Severe hypertonia and multiple contractures significantly limit gross motor development among the infants in the study. Most children in this sample were reported to push up in prone (72%; precursor to crawling) and stand with support (55%; precursor to walking); however, only one child was reported to be able to sit unsupported or walk with support. Around one-quarter were able to roll from supine to prone and/or maintain a crawling position on their hands and knees. Males scored significantly higher than females on gross motor skills (0.34, p < .05).

### Fine motor skills

Fine motor skills were reported to be significantly limited across the sample. Many of the children had difficulty opening their hands from a fisted position. Less than half were able to hold a small object in their hands and less than one-quarter were able to obtain objects with their fingers or bring their hands to their mouths. The severity of visual impairment was significantly associated with fine motor skills (-0.350, p < .016).

### Problem solving

Around one-quarter of infants in this study had mastered reaching for toys with two hands and exploring those toys with their mouths. Around 10% were able to play with toys at a table or on the floor and demonstrated early object permanence by looking for a dropped toy. Only one or two were able to pass toys from hand to hand, hold more than one toy, or bang toys in play.

### Personal-social

Around one-third of the babies in this sample demonstrated social interest—smiling or making sounds at a mirror and differentiating between familiar and unfamiliar people. Slightly fewer than half of the babies were able to drink liquids from a cup held by a caregiver.

### Sleep

Sleep was not reported be a problem for the majority (79%) of infants. On average, the infants were reported to be sleeping around 9 hours at night (SD = 2.3; range = 3–16 hours) and 2.3 hours during the day (SD = 2; range 20 min–9 hours). Latency to fall asleep was, on average, around 32 minutes (SD = 32.1; range 5–120 minutes).

### Conclusions

Infants in Brazil whose mothers were infected with ZIKV during pregnancy have captured the world’s attention due to the severity of birth defects seen in many affected children. The long-term developmental outcomes for these infants are unknown but are thought to be severe due to the known brain and central nervous system dysfunctions that have been documented in early cohorts of affected infants. However, these children will continue to grow and develop, and understanding what developmental skills they are able to obtain is critical to providing the appropriate types and doses of intervention as they age.

This study represented a preliminary assessment of discrete developmental skills in 47 babies with documented CZS. The study found that, at 16 months of age, these children were all functioning substantially below developmental expectations; most are estimated to be well under 6 months of age developmentally. However, profiles of developmental strengths and weaknesses did emerge with a closer look at the subdomains and items on the ASQ-3. Specifically, the children appeared to have relative strengths in communication and gross motor skills, while weaknesses were found in fine motor skills and other skills requiring fine motor.

Within the communication domain, most infants seemed to respond to their environment through sound. Given the extent of visual impairment and motor planning issues seen in these children thus far [4], auditory stimulation is likely, at least initially, to be their primary means for exploring their environment. Also, the majority of the infants were able to differentially recognize familiar voices, suggesting that they may also benefit from increased social interactions. Whether communication remains a relative strength over time will depend in part on the type and amount of stimulation the child receives from others in their environment as well opportunities for alternative means of communication. These findings point to the importance of interventions that focus on increasing verbal exchanges between caregivers and infants and identifying methods by which the children can effectively communicate their wants and needs.

Gross motor skills were also a relative strength for the sample; however, only one child had reached basic early motor milestones such as controlled, independent sitting or walking with support. Some of the early precursor skills that were endorsed by parents—such as pushing up in prone or standing with support—may not, in fact, be due to developmental attainment of those skills but rather to the effect of the extreme hypertonia many of these children experience. In other words, they may be able to remain in a position in which they are placed (prone or standing) because their muscles are so tight and contracted, not because they can maintain those positions intentionally in an effort to become more mobile (i.e. to crawl or walk).

Fine motor skills were a relative weakness for this sample. While around one-third of the infants were able to hold an object in their hands, very few were using both hands or manipulating the objects in any way. As with gross motor skills, hypertonia and contractions likely interfere with fine motor development such that the infants can hold the items because their hands contract around the object, but are unable to release or manipulate them. These fine motor challenges also potentially interfere with their ability to display problem-solving skills, which require increasingly more complex motor planning as the children get older.

Sleep did not appear to be a concern for most of the infants in this study. The percentage of infants in our sample who had difficulty with sleep, the reported number of hours the infants slept at night and during the day, and the amount of time it took for them to fall asleep all resembled a larger sample of typically developing infants in Brazil [11]. However, these findings contrast with reports of sleep issues in a larger sample of children with CZS reported by Pinato and colleagues [12], who found that the children with CZS were significantly more likely to be reported as having sleep problems and having shorter total sleep time and shorter nocturnal sleep duration that a typically developing control group. These differences in findings may be due to differences in sample size between the two studies. Another explanation may be that the Pinato study had a much larger age range (5–24 months), which included very young infants, who typically have more sleep problems that resolve as they get older. Indeed, Pinato et al found that almost half of the children with CZS who were assessed more than once had increased their sleep durations as they got older. Sleep electroencephalogram patterns of infants with CZS have been reported to be abnormal, even among those without seizures [13], suggesting that the quality of sleep the infants are getting may be poorer than caregivers can ascertain by observation. As poor quality of sleep can further impact development, ongoing monitoring of sleep quality and quantity is needed.

Although our sample size precludes our ability to draw conclusions about what variables might be predictive, the correlations between the family and individual factors and the outcomes provide some positive direction for future research. For example, the severity of visual impairment was associated with fine motor skills. Research on early brain development suggests that visual and hearing pathways develop first and facilitate the development of communication and cognitive functions [14]. Visual impairment limits access to social and environmental stimuli that are critical for problem-solving and personal social development. However, these findings could also reflect a third variable, such as documented brain abnormalities that impact both visual and motor planning. Future research should certainly include the severity of visual impairment as well as response to visual therapy. In addition, it will be important to further examine imaging findings to determine if there are brain-based features that could be predictive of better or worse outcomes. Further, while we were able to obtain information about much of the participants medical history, we are missing information on several important features, including the incidence of hydrocephaly and related shunt surgeries, as well as prevalence and severity of seizures, important co-occurring medical needs that should be explored in future research.

Interestingly, in our study, males as a group performed better on all domains of development than did females, partly because two infants who had obtained earlier developmental milestones were male, thereby skewing the male infant data. However, even with those outliers removed from the sample, males outperformed females on gross motor and fine motor skills, with almost twice the number of points, on average, on each scale. Sex-based differences in performance over time will be an important trend to monitor.

No significant associations were found between developmental outcomes and the severity of microcephaly or timing of ZIKV exposure, factors thought to be important for predicting outcomes. However, given that most cases had severe microcephaly and were believed to have been exposed to ZIKV in the first or second trimester, there may not be enough variability within these factors to detect associations. Instead, factors such as birthweight and other exposures in utero seemed to have stronger associations. These findings reflect the importance of considering cumulative exposures in determining long-term outcomes for these infants.

This study has some clear limitations. The sample size was small, limiting our ability to generalize to the larger population of infants with CZS and precluding our ability to more comprehensively examine potential predictors of outcomes. This study also lacks a comparison group, which limits our ability to make causal statements. However, the description of developmental skill attainment is based on a norm-referenced measure—the ASQ-3; which allows us to estimate a rough developmental level based on the skills mastered or emerging for each child. However, the ASQ-3 was challenging to use for several reasons. It includes different age-based forms and clinicians had difficulty determining which form was most appropriate for which infant, especially given that none of the infants were able to demonstrate skills at their chronological age level. Further, all of our assessments were based on parent report, which may have been biased or at least limited by the parent’s comprehension and interpretation of the items.

However, despite these limitations, this study provides valuable information about discrete developmental skills obtained by infants with CZS within the first 18 months of life. This is the first glimpse into developmental strengths and weaknesses in children with CZS, which can provide a foundation for monitoring developmental trajectories. We have subsequently initiated a 5-year longitudinal prospective study, using multiple methods for comprehensive assessment of 200 children and factors that may impact their development. Given the devastating and lifelong nature of CZS, longitudinal research that addresses and supports the needs of the developing child and their families will be essential.
